# Stimulus-category competition, inhibition, and affective devaluation: a novel account of the uncanny valley

**DOI:** 10.3389/fpsyg.2015.00249

**Published:** 2015-03-13

**Authors:** Anne E. Ferrey, Tyler J. Burleigh, Mark J. Fenske

**Affiliations:** ^1^Child Study Center, Yale University School of Medicine, New HavenCT, USA; ^2^Department of Psychology, University of Guelph, GuelphON, Canada

**Keywords:** uncanny valley, cognitive conflict, inhibition, affect, emotion, inhibitory devaluation, visual perception, cognitive dissonance

## Abstract

Stimuli that resemble humans, but are not perfectly human-like, are disliked compared to distinctly human and non-human stimuli. Accounts of this “Uncanny Valley” effect often focus on how changes in human resemblance can evoke different emotional responses. We present an alternate account based on the novel hypothesis that the Uncanny Valley is not directly related to ‘human-likeness’ *per se,* but instead reflects a more general form of stimulus devaluation that occurs when inhibition is triggered to resolve conflict between competing stimulus-related representations. We consider existing support for this inhibitory-devaluation hypothesis and further assess its feasibility through tests of two corresponding predictions that arise from the link between conflict-resolving inhibition and aversive response: (1) that the pronounced disliking of Uncanny-type stimuli will occur for any image that strongly activates multiple competing stimulus representations, even in the absence of any human-likeness, and (2) that the negative peak of an ‘Uncanny Valley’ should occur at the point of greatest stimulus-related conflict and not (in the presence of human-likeness) always closer to the ‘human’ end of a perceptual continuum. We measured affective responses to a set of line drawings representing non-human animal–animal morphs, in which each continuum midpoint was a bistable image (Experiment 1), as well as to sets of human-robot and human-animal computer-generated morphs (Experiment 2). Affective trends depicting classic Uncanny Valley functions occurred for all continua, including the non-human stimuli. Images at continua midpoints elicited significantly more negative affect than images at endpoints, even when the continua included a human endpoint. This illustrates the feasibility of the inhibitory-devaluation hypothesis and the need for further research into the possibility that the strong dislike of Uncanny-type stimuli reflects the negative affective consequences of cognitive inhibition.

## Introduction

The Uncanny Valley— a significant decrease in liking for objects that closely resemble humans but are not perfectly human-like— was originally described in terms of the uncomfortable feeling associated with viewing robots of increasing human-likeness (Mori, 1970). Indeed, many accounts of this effect have focused on the potential relationship between the subjective human-likeness of a stimulus and an observer’s emotional response to it (e.g., MacDorman and Ishiguro, 2006; Seyama and Nagayama, 2007; MacDorman et al., 2009). The importance of elucidating specific mechanisms underlying the Uncanny Valley effect is underscored by the extent to which interest in this effect has spread from robotics into other areas, such as computer graphics and prosthetics (Seyama and Nagayama, 2007; MacDorman et al., 2009; Mitchell et al., 2011; Tinwell et al., 2011; Poliakoff et al., 2013). To this end, we consider here the novel hypothesis that the Uncanny Valley is not directly related to ‘human-likeness’ *per se,* but instead reflects a more general form of stimulus devaluation that occurs when inhibition is triggered to resolve conflict between competing stimulus-related representations. The purpose of this article is to therefore demonstrate how the Uncanny Valley may be explained through recent advances in our understanding of the negative affective consequences of cognitive inhibition. After presenting our ‘inhibitory-devaluation’ hypothesis, we report a preliminary assessment of its feasibility—in terms of prior findings as well as through two new experiments that test specific predictions arising from this new account of the Uncanny Valley—and then consider directions for future research.

### Inhibition, Negative Affect, and the Uncanny Valley

A recent and growing body of research suggests that cognitive inhibition is not only crucial for resolving potential interference during visual tasks (i.e., when multiple stimulus/response representations compete to become the focus of thoughts and actions), but also subsequently results in negative affect for the associated stimuli (for reviews, see Fenske and Raymond, 2006; Raymond, 2009). Such affectively negative consequences of inhibition have been found in a variety of visual-recognition tasks that require stimulus classification (e.g., Kiss et al., 2008; Frischen et al., 2012) and localization (e.g., Raymond et al., 2003; Fenske et al., 2004), using stimuli ranging from meaningless patterns (e.g., Raymond et al., 2003), non-human objects (e.g., Griffiths and Mitchell, 2008), and entire scenes (Frischen et al., 2012), to images of real human faces (Fenske et al., 2005), and bodies (Ferrey et al., 2012). Moreover, these studies have shown that this inhibitory devaluation impacts a variety of subjective emotional judgments (i.e., likeability, relative preference, cheerfulness, pleasantness, trustworthiness, sexual attractiveness), as well as the motivational incentive to seek and obtain otherwise-appealing stimuli. Importantly, the magnitude of inhibitory devaluation increases with the level of potential interference from competing stimulus-category or stimulus-response representations (e.g., Raymond et al., 2005; Frischen et al., 2012; Martiny-Huenger et al., 2013). This suggests that the Uncanny Valley effect could be a specific instance of inhibition-related devaluation of stimuli whose perception activates multiple, competing stimulus interpretations.

Most prior accounts of the Uncanny Valley effect suggest it occurs when humans view images of conspecifics (i.e., other potential humans) that possess non-human traits. For instance, one idea is that disliking of not-quite-humanlike images is the result of a disgust response that evolved for the purpose of pathogen avoidance (MacDorman and Ishiguro, 2006; MacDorman et al., 2009). From this perspective, the stronger an entity’s resemblance to a conspecific, the stronger the aversion to a “deformed” version would be, since defects may cue potential disease, and conspecific resemblance cues the potential for catching the disease due to genetic similarity (Rozin and Fallon, 1987). By extension, negative feelings elicited during studies that have used human-like stimuli with mismatched features (e.g., Seyama and Nagayama, 2007; MacDorman et al., 2009; Mitchell et al., 2011) could reflect the activation of this human-specific pathogen-avoidance mechanism.

In contrast to theories focusing on discrepancies related to the ‘human-ness’ of stimuli, an inhibitory-devaluation account of the Uncanny Valley predicts that negative evaluations will be triggered by any stimulus that activates multiple, competing stimulus representations during recognition. Recent reviews of the neurocognitive mechanisms underlying the perception of ambiguous sensory information suggest that resolving such competition, and the accompanying perceptual ambiguity, may be achieved through mechanisms that suppress neural activity associated with perceptual features that conflict with the perceptual outcome (e.g., Sterzer et al., 2009). This perspective is consistent with well-accepted biased-competition models of visual processing (e.g., Desimone and Duncan, 1995), whereby each possible interpretation of an ambiguous figure competes for representation across a hierarchical network of visual areas. Top–down signals, such as those associated with selective attention, can bias this neural competition in favor of one perceptual interpretation over another (Meng and Tong, 2004). Evidence that inhibition of competing representations may be one of the mechanisms through which the competition is biased to resolve such conflict (e.g., Munakata et al., 2011) suggests that the well-established negative affective consequences of inhibition should be evident for any stimulus that activates multiple, competing stimulus representations during recognition, just as it is for stimuli associated with other forms of inhibition (Fenske and Raymond, 2006; Raymond, 2009).

Support for a shift in focus from human-specific to more general recognition-related mechanisms can also be seen in recent ‘categorization’ accounts of the Uncanny Valley (Cheetham et al., 2011; Moore, 2012; Burleigh et al., 2013; Cheetham et al., 2013; Burleigh and Schoenherr, 2015). These accounts generally consider affective response to be a function of stimulus distance from a category boundary (Cheetham et al., 2011, 2014; but see Burleigh and Schoenherr, 2015). A stimulus is easy to classify as ‘human’ or ‘non-human’ when it is far from the category boundary along a ‘human’/‘non-human’ continuum. But a stimulus that is at or near the category boundary is difficult to classify because its identity is ambiguous. Cheetham et al. (2011, 2014) provided evidence of this category boundary at the midpoint of a human-avatar morph continuum, and Burleigh et al. (2013) reported that ambiguous morph-stimuli near the midpoint of a human–non-human continuum were indeed associated with heightened levels of negative affect. The findings of Cheetham et al. (2014) further support the idea that such devaluation may be linked to categorization, but is not likely to follow from perceptual discrimination difficulty, *per se*. Importantly, these previous results are consistent with the possibility that stimuli near such midpoints strongly activate multiple, competing visual-category representations during recognition. From this perspective, negative affect for such items occurs to the extent that selecting one interpretation over the other requires inhibition of the visual-category information associated with the non-selected interpretation. The greater the inhibition during identification, the greater the negative affect for the associated stimulus.

A key feature of Uncanny Valley explanations that focus on the ‘human-ness’ of stimuli concerns the expected location of the ‘valley’ – the point along a perceptual continuum where stimulus-related affective response maximally deviates from an otherwise linear function. A conspecific pathogen-avoidance account, for example, predicts an asymmetrical valley that drops closer to the ‘human’ side of a ‘human’/‘non-human’ continuum. Indeed, this is exactly what was depicted in Mori’s (1970) well-known original figure illustrating the Uncanny Valley. In contrast, an inhibitory-devaluation account predicts the greatest affective drop at the point where multiple competing visual-category representations are most strongly activated. And while this should be at the midpoint for continua anchored by two equally distinct stimulus categories, the exact location for any given continuum will vary depending on parameters such as baseline affective response to the specific endpoints and the perceptual salience of the visual cues denoting each category. This may explain why Seyama and Nagayama (2007, Experiments 3), for example, were able to obtain a valley location comparable to that depicted by Mori (1970), but only after dramatically increasing the size (and thus the perceptual salience) of the discrepant features in otherwise highly human-like stimuli. Other studies utilizing ‘human’/‘nonhuman’ continua have found valleys at the midpoint, and occasionally closer to the ‘non-human’ endpoint (MacDorman and Ishiguro, 2006; Burleigh et al., 2013). Such findings are consistent with the possibility that the lowest point in the valley is not determined by the human-ness of the stimuli *per se*, but instead occurs at whatever point requires the greatest inhibition of competing visual-category information to select one stimulus interpretation over another.

Inhibition has been proposed as a critical mechanism for resolving conflict and potential interference from competing signals in a variety of cognitive and neural operations (for review, see Munakata et al., 2011). And while evidence of the link between conflict-resolving inhibition and negative affect has only recently begun to accumulate, several lines of research, including Burleigh et al.’s (2013) examination of the Uncanny Valley, have demonstrated the link between situations involving cognitive conflict and negative affect. A classic example is cognitive dissonance theory, which originally described how a negative emotional response is elicited when a person’s attitude is at odds with their behavior (Festinger, 1957). In more general terms, cognitive dissonance describes a conflict between two incompatible cognitions (van Veen et al., 2009), which leads to both autonomic arousal and negative affect (Croyle and Cooper, 1983; Losch and Cacioppo, 1990; Elliot and Devine, 1994) and has been described as a state of discomfort and unease (Elliot and Devine, 1994). In order to resolve this conflict, cognitive resources must be devoted to the problem. The corresponding negative affect may be linked in part to the extent that the resources recruited in the face of such conflict include inhibition aimed at reducing the salience of incompatible representations.

A special case of cognitive dissonance is *post-decisional dissonance.* This phenomenon was first observed in Brehm (1956), who noticed that participants who were asked to choose between two similarly valued items had rated selected items more positively than their initial ratings of the same item, and rated the rejected item more negatively. In this case, a conflict between two choices lead to negative affect associated with the unchosen item. This may result from recruitment of cortical areas that inhibit representations of the unchosen option, leading to negative affect (Harmon-Jones, 2004).

Other types of interference or conflict are also associated with negative affect. For example, the interference that is caused by response competition during the Stroop (1935) task when the name of a color is presented in a color that is inconsistent with its identity (e.g., the word “blue” in red ink). When conflict occurs, the dorsal anterior cingulate cortex (dACC; Botvinick et al., 2001; van Veen et al., 2009; Izuma et al., 2013) and insula regions (van Veen et al., 2009) are activated, and individuals experience arousal and feelings of discomfort (Elliot and Devine, 1994; van Veen et al., 2009), which motivates them to engage in a dissonance-reduction strategy. In the case of a Stroop (1935) task, for example, dissonance-reduction is accomplished by biasing inputs such that word names or word colors dominate response selection (Botvinick et al., 2001).

The consistency of prior findings with what we have recently learned about the affective consequences of inhibition suggests that an inhibitory-devaluation account of the Uncanny Valley effect merits further consideration. One way to further assess its feasibility is to begin experimentally testing specific predictions that arise from our account. We report two such tests below as a preliminary example of Uncanny Valley research into the inhibitory-devaluation hypothesis.

## Assessing the Feasibility of the Inhibitory-Devaluation Hypothesis

The hypothesis that the Uncanny Valley reflects inhibitory devaluation of stimuli that activate multiple, competing stimulus interpretations during recognition generates a number of testable predictions. The two considered here concern the type of stimuli that can show Uncanny Valley effects and the type of stimuli that are likely to receive the most negative affective evaluations. Our experimental approach for assessing the potential involvement of cognitive inhibition in the Uncanny Valley is the same as that used extensively in studies of inhibitory devaluation. In these prior studies, any type of stimulus—human or non-human— appearing in experimental conditions suspected to involve cognitive inhibition have consistently received more negative evaluations than stimuli appearing in conditions thought to be relatively free of inhibition (Fenske and Raymond, 2006; Raymond, 2009). Such effects are routinely obtained across substantially different visual classification and response-decision tasks. Indeed, the link between inhibition and stimulus devaluation is sufficiently strong that researchers have begun to take the occurrence of increasingly negative subjective stimulus evaluations as a key indicator of the potential involvement of inhibition at key points within a given task (e.g., Kihara et al., 2011). Thus, while we do not directly measure inhibition *per se*, we instead assess whether differences in affective evaluations for items at different points along a given perceptual continuum are consistent with the expected extent to which inhibition may be applied during the perception of such items. The experiments reported in this section therefore represent an important first step in assessing the feasibility of an inhibitory-devaluation account by (1) confirming that the Uncanny Valley also occurs when humans view distinctly non-human stimuli, and (2) demonstrating that for human-like stimuli, the lowest point of the ‘valley’ does not always occur on the ‘human’ side of the perceptual continuum.

The key emphasis on the human-ness of objects in prior accounts of the Uncanny Valley effect may explain why so little work has been done to explore whether Uncanny Valley-type effects also occur with non-human stimuli. To the best of our knowledge, the only published research of this sort currently includes two papers by Yamada et al. (2012, 2013). However, their findings do suggest that the Uncanny Valley can occur for non-human stimuli. Yamada et al. (2012) obtained participants’ categorization responses and affective ratings of images that morphed between a tomato and a strawberry. The lowest likability scores for these images coincided with the point of greatest ambiguity in stimulus categorization. Yamada et al. (2013) likewise found that participants’ most affectively negative ratings were provided for the most ambiguous stimuli among sets of images that morphed between a real dog and a cartoon dog, a real dog and a stuffed-toy dog, and a cartoon dog and a stuffed-toy dog. Unfortunately, the veracity of these findings remains unclear because of issues associated with the use of relatively small sample sizes (e.g., Yamada et al., 2013, utilized 12 or fewer participants in each experiment), and the possibility that their stimuli included visual artifacts produced by the morphing process that may have had a confounding influence on participants’ affective ratings.

Our studies therefore expand upon these important prior findings to provide a converging test of the prediction that Uncanny Valley effects should not be limited to the perception of humans, but should also occur with non-human stimuli. Thus, in Experiment 1, we use bistable line drawings of animals, a type of non-human stimuli that has been specifically designed to activate multiple, competing stimulus interpretations (Fisher, 1967). The sets of line-drawn images we used were specifically chosen for their step-wise differences along a given two-category continuum and because of the availability of normative data regarding the corresponding level of perceptual ambiguity of each item (Verstijnen and Wagemans, 2004).

Accordingly, the conditions in Experiment 1 in which we suspect the greatest involvement of inhibition during perception concern those items whose perceptual features are consistent with multiple conflicting interpretations, such as those near the midpoint of a given continuum. In contrast, recognition of items closer to a continuum endpoint, whose perceptual features are clearly consistent with a single interpretation, should be relatively free of inhibition. Following the experimental approach of prior inhibitory devaluation studies, we therefore expect that any stimulus occurring near the midpoint of such a two-category continuum should receive more negative evaluations than items near the continuum endpoints. Participants in our studies were asked to evaluate stimuli based on their initial emotional reaction to each stimulus using a numerical rating scale. This allowed us to obtain an accurate measure of subjective emotional reactions to non-human stimuli that vary in the extent to which they activate multiple, competing stimulus interpretations.

In addition to responses to non-human stimuli, we also predicted that when assessing affective responses to human-like stimuli, the location of the ‘valley’— the point along a perceptual continuum where stimulus-related affective response maximally deviates from an otherwise linear function — should occur near the midpoint of a two-category continuum. We tested this prediction in Experiment 2 by examining differences in individuals’ affective responses to sets of 3D computer-modeled images that represent different points along human-to-robot and human-to-animal morphed continua.

## Experiment 1: Non-Human Bistable Images

### Materials and Methods

**FIGURE 1:**
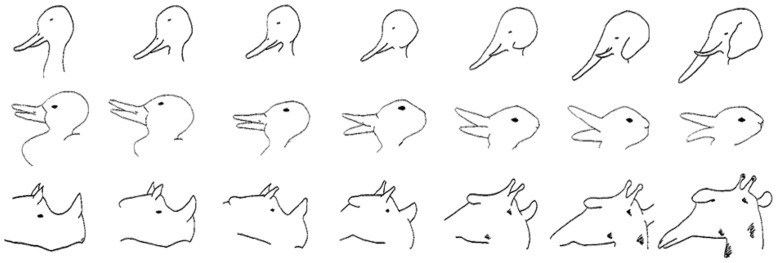
**Bistable image examples (rabbit-duck, rhino-giraffe, and duck-elephant; originally from Hogeboom (1995), as cited in Verstijnen and Wagemans (2004)**.

Stimuli in this experiment consisted of three different sets of line drawings, each comprising a step-wise continuum of differences in perceptual similarity to two distinct animals. The stimulus at the midpoint of each continuum is a bistable image that can be interpreted as either of the two animals (see **Figure 1** for example). Bistable images have long been of interest (e.g., Fisher, 1967) as stimuli that can support two incompatible interpretations, although the stimulus itself does not change. Such stimuli are specifically created to ensure maximal category conflict for items near the bistable midpoint. Normative stimulus-classification data provided by Verstijnen and Wagemans (2004) previously established that the point of maximal perceptual ambiguity for these stimulus sets was for the item within one step of the midpoint (i.e., midpoint plus or minus one step) of each continuum.

We predicted that the shape of the affective data for stimuli from each continuum would be consistent with an Uncanny Valley function (i.e., non-linear). Following Burleigh et al. (2013), we tested this by fitting linear, cubic, and quadratic functions to the data for each continuum. Because the Uncanny Valley function (Mori, 1970) is essentially a cubic function, we expected a cubic or quadratic function would fit our data better than a linear function. We also predicted that the specific shape of this non-linear function would be formed by lower affective ratings for morph-stimuli near the midpoint of the continua than for those near the endpoints.

All of the following materials and procedures were approved by the Research Ethics Board at the University of Guelph (REB #11NV011).

#### Participants

Sixty undergraduate students (31 women, *M_age_* = 20 years, SD_age_ = 3.6) participated in exchange for course credit. The only inclusion criterion was having normal or corrected-to-normal vision.

#### Apparatus and stimuli

Stimuli consisted of three sets line drawings, each comprising seven images (Hogeboom, 1995, as cited in Verstijnen and Wagemans, 2004). Sets included: duck-elephant, rhino-giraffe, and rabbit-duck (see **Figure 1**).

The 21 images were presented in a randomized order for each participant using an Intel Core2Duo computer with a 50.8 cm LCD monitor (resolution: 1680 × 1050 pixels) running PsychoPy software (Peirce, 2007). Displays were viewed at a distance of 75 cm in a sound-attenuated room, with low ambient illumination. Stimuli were presented one at a time at the center of the screen for 400 ms along with a visual-analog rating scale that ranged from “dislike very much” (0.00) to “like very much” (1.00). Participants were required to use the mouse to select the point on the line that best matched their emotional response to each stimulus. The rating scale had a precision of 0.01 unit increments, and was visible on the screen until a response was registered.

### Results

**FIGURE 2:**
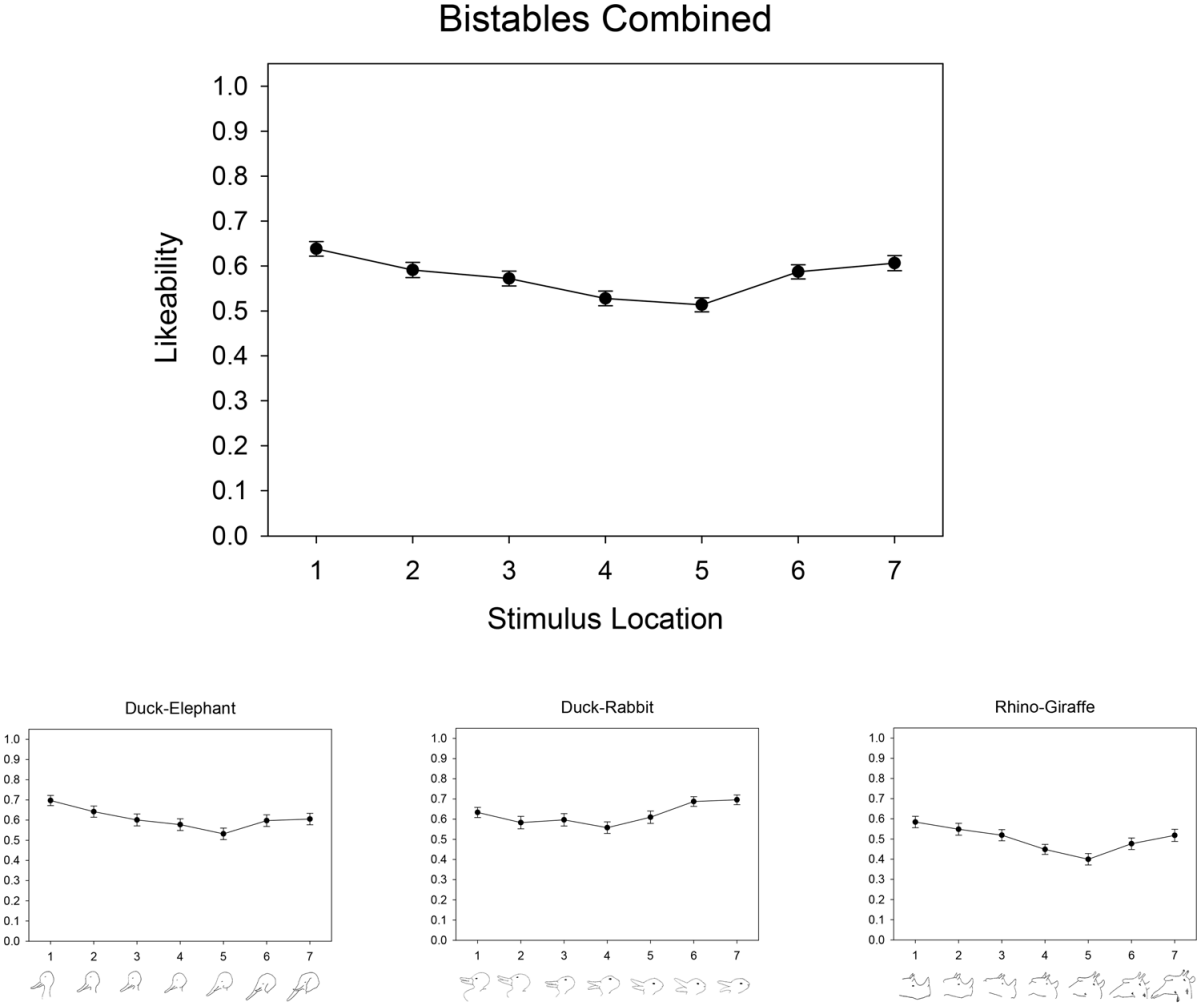
**Affective trends for animal-animal bistable morphs in Experiment 1**.

Average ratings were calculated for each stimulus as a function of its location along its corresponding continuum. These are plotted separately for each stimulus set in **Figure 2**, along with average ratings calculated across the three stimulus sets.

We predicted that the shape of the affective data for stimuli from each continuum would be consistent with an Uncanny Valley function (i.e., non-linear). To assess this, we fit linear, cubic, and quadratic functions to the data for each continuum, as in Burleigh et al. (2013). Because the Uncanny Valley function (Mori, 1970) is essentially a cubic function, it follows that if a cubic or quadratic function was found to fit the data better than a linear function, then this would support an Uncanny Valley interpretation.

We used the Akaike Information Criterion (AIC; see Burnham and Anderson, 2002) as our goodness-of-fit index. The AIC is suited to comparing models with different degrees of complexity because it penalizes models with additional fit parameters. We calculated raw Akaike values and Akaike Weights (*w_i_*), which are a transformation of raw scores that indicate the probability that a particular model among the set of models is correct (Wagenmakers and Farrell, 2004). Using these weights, we also calculated evidence ratios by dividing the weight of one model by the weight of another. These ratios are understood in context of a “confidence set,” which is similar to a confidence interval and is defined as 10% of the highest Akaike Weight in the set (Royall, 1997). For the purposes of interpretation, it should be noted that lower raw Akaike values and higher Akaike Weights indicate a better fit to the data.

As indicated by the evidence ratios in **Table 1**, our curve-fit analyses confirmed that non-linear quadratic and cubic models were best fit to the data, whereas linear models fell outside the confidence set. To the extent that such non-linear functions are a defining feature of the Uncanny Valley (Burleigh et al., 2013), this finding is consistent with the possibility that Uncanny Valley effects can occur with distinctly non-human stimuli.

**Table 1:** Experiment 1 curve fit analyses.

Set	Model	*Residual sum of squares*	*AICc*	*Δ_i_(AIC)*	*w_i_(AIC)*	*CI*
Duck-elephant*	Linear^1^	20.31	-1270.26	7.66	0.02	0.07
	Quadratic^2^	19.85	-1277.92	0.00	0.71	–
	Cubic^3^	19.84	-1276.03	-1.89	0.28	–
Rabbit-duck*	Linear	19.92	-1278.45	7.62	0.01	0.07
	Quadratic	19.47	-1286.07	0.00	0.65	–
	Cubic	19.44	-1284.71	1.35	0.33	–
Rhino-giraffe*	Linear	20.76	-1261.06	12.91	0.00	0.07
	Quadratic	20.10	-1272.67	1.29	0.34	–
	Cubic	19.94	-1273.96	0.00	0.66	–

^1^*K* = 1, ^2^*K* = 2, ^3^*K* = 3, *n = 420.

Indeed, we predicted that the specific shape of this non-linear function for bistable images would further resemble an Uncanny Valley by having lower affective ratings for morph-stimuli near the midpoint of the continua than for those near the endpoints. The average rating for stimuli at positions 1 and 7—the unambiguous category endpoints—was therefore compared to the average rating for the position-4 midpoint stimulus for each stimulus set using paired-samples *t*-tests. Consistent with our expectations, endpoint items were rated more positively than midpoint items from the duck-elephant [endpoints, *M* = 0.65, SD = 0.17; midpoint, *M* = 0.58, SD = 0.22; *t*(59) = 2.86, *p* = 0.006], rabbit-duck [endpoints, *M* = 0.66, SD = 0.16; midpoint, *M* = 0.56, SD = 0.22; *t*(59) = 4.64, *p* < 0.001], and rhino-giraffe [endpoints, *M* = 0.54, SD = 0.17; midpoint, *M* = 0.45, SD = 0.18; *t*(59) = 3.80, *p* < 0.001] sets.

**FIGURE 3:**
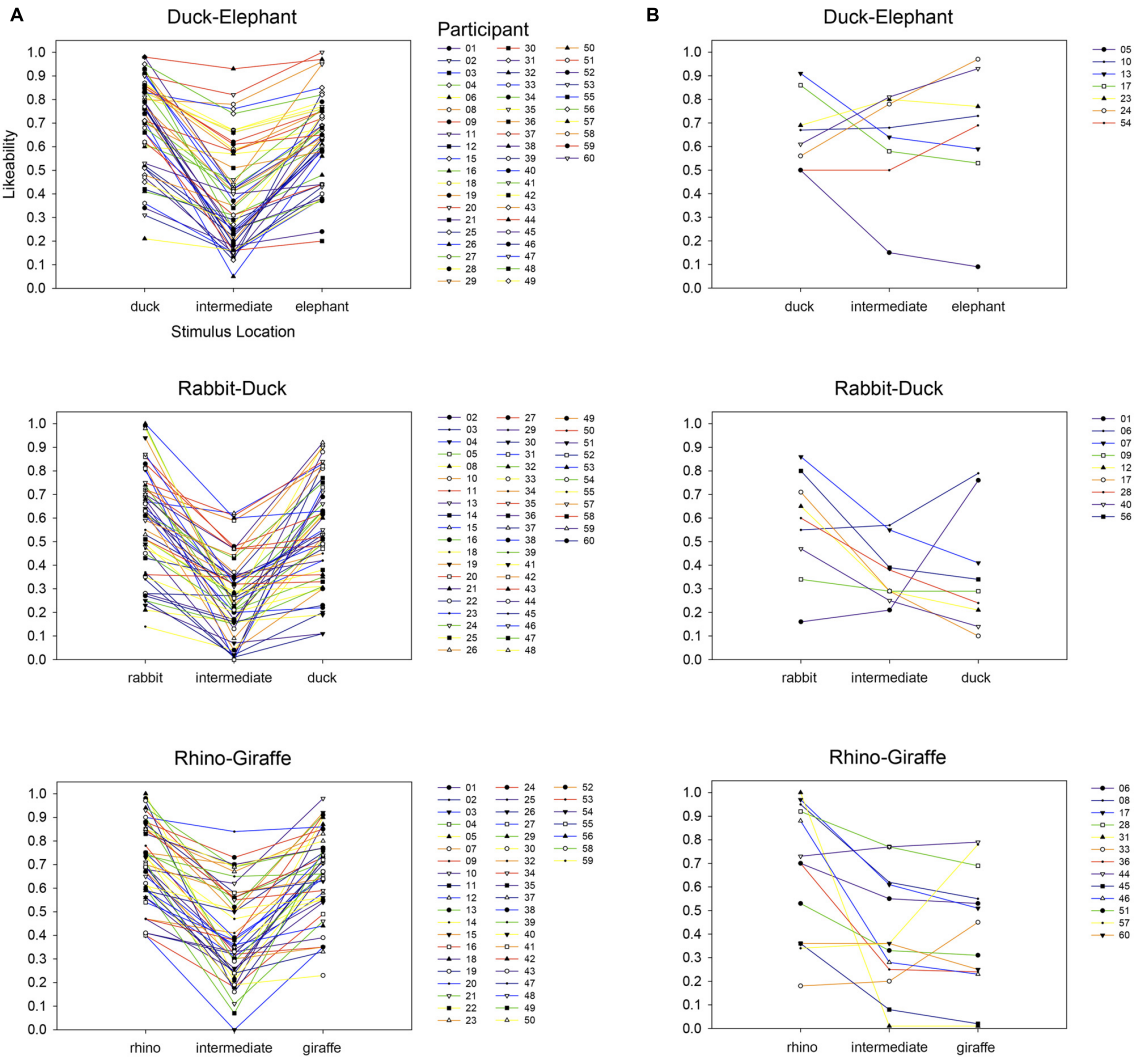
**Individual participants’ affective response to each animal–animal bistable morph continua in Experiment 1.** Abbreviated rating functions are each comprised of a specific participant’s rating of the stimulus at each endpoint (i.e., positions 1 and 7) along with the lowest rating they provided to an intermediate stimulus (i.e., among positions 2–6). The majority of participants **(A)** provided their lowest affective rating to a stimulus from an intermediate position. The resulting ‘valley’ shape of these individual rating functions is consistent with the Uncanny Valley-type effects identified in the group-average results, but is absent in a minority of participants **(B)** who each provided their lowest rating to at least one of the continua-endpoint items.

Our results based on average ratings suggest that participants provided their lowest affective ratings to morph-stimuli at intermediate positions of each continuum rather than to those at either continuum endpoint. To examine the extent to which this pattern was observable at the level of individual participants, we plotted an abbreviated rating function for each participant’s affective response to each perceptual continuum. This was comprised of each participant’s rating of the stimulus at each endpoint (i.e., positions 1 and 7) along with the lowest rating they provided to an intermediate stimulus (i.e., among positions 2–6). As shown in **Figure 3A**, for each perceptual continuum, the vast majority of participants (85% for duck-elephant, 87% for duck-rabbit, 80% for rhino-giraffe) provided their most negative affective rating in response to an intermediate stimulus. The resulting ‘valley’ shape of these individuals’ rating functions is visually evident despite substantial variability otherwise in their individual responses. However, it is also the case that, for each perceptual continuum, a corresponding minority of participants provided their lowest rating for an endpoint stimulus, failing to show a valley shape in their individual rating functions (see **Figure 3B**). This suggests that while most individuals’ affective responses were appropriately reflected by our group averages, others do not show the same Uncanny-valley-type pattern of responses (see MacDorman and Entezari, 2015, for another example of individual differences in Uncanny Valley effects).

Taken together, the results of Experiment 1 replicate and expand upon the important preliminary findings of Yamada et al. (2012, 2013) to confirm that Uncanny Valley-type effects can occur with distinctly non-human stimuli. These findings are therefore consistent with the possibility that the drop in affective response reflected by the Uncanny Valley may not be determined by the human-ness of the stimuli *per se*, but might instead occur at whatever point requires greatest inhibition of competing visual-category information to select one stimulus interpretation over another.

## Experiment 2: 3D Computer Models

### Materials and Methods

The possibility that the Uncanny Valley reflects inhibitory devaluation of stimuli that activate multiple, competing stimulus interpretations during recognition suggests that the location of the valley—the continuum point showing the most affectively negative stimulus response— should occur at the point where multiple competing visual-category representations are most strongly activated. Thus, in contrast to explanations that focus on the ‘human-ness’ of stimuli, this lowest point for human-like stimuli should not always occur on the ‘human’ side of the perceptual continuum.

To test this prediction, we measured affective responses to a series of 3D computer-modeled morph stimuli representing different locations along different human-non-human continua. To replicate the classic Uncanny Valley effect, one stimulus set was created from human-robot morphs. Additional stimulus sets were created from various human–animal continua. For all stimulus sets, we expected stimuli near continua midpoints to receive more negative affective ratings than those depicting category endpoints. Furthermore, we predicted that the greatest drop in affective ratings would not consistently occur at stimulus-continuum locations near the “human” endpoint, as predicted by a conspecific pathogen-avoidance account of the Uncanny Valley effect.

All of the following materials and procedures were approved by the Research Ethics Board at the University of Guelph (REB #11NV011).

#### Participants

Sixty-nine undergraduate students (54 women, *M_age_* = 19 years, SD_age_ = 1.2) participated in exchange for course credit. The only inclusion criterion was having normal or corrected-to-normal vision. None of the participants in Experiment 2 had previously participated in Experiment 1.

#### Apparatus and stimuli

**FIGURE 4:**
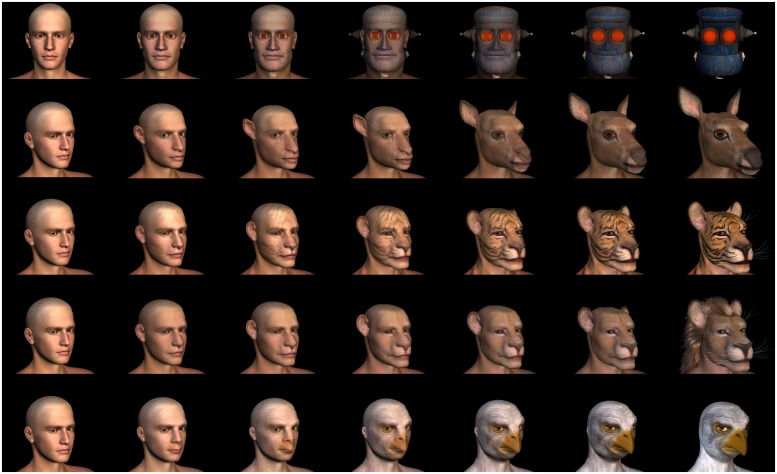
**Computer-generated morphs (human-robot, human-stag, human-tiger, human-lion, human-bird)**.

Stimuli consisted of 35 computer-generated images that were created using Poser (Version 2012, www.smithmicro.com) modeling software and Abrosoft FantaMorph (Version 5.4, www.fantamorph.com) morphing software. In all, there was one human-robot morph-continuum, and four human-animal continua (human-stag, human-tiger, human-lion, and human-bird), each with seven continuum levels – see **Figure 4**.

In order to create the human-animal stimuli, a “base” human model, *Michael 4*, was obtained from daz3d.com, and modified using commercial morph packages for Poser (specifically, the *Leonese* and *Cervus* characters obtained from philosophersegg.com, and the* Bird Cult* character obtained from daz3d.com). Each of these morph packages comprise a pre-defined set of morphological transformations that can be applied to a base model in order to holistically transform its morphology into the animal character. These packages also contain textures that transform the “skin” of the character into the fur of the animal. A crucial aspect of these morph packages is that the transformations can be applied in a continuous fashion, by assigning values between 0.000 and 1.000 (e.g., a transformation value of 0.500 would be morphologically half-human and half-animal). Similarly, textures can be applied in a continuous fashion, by applying both the human and animal textures to the same figure, and setting them to different levels of opacity (e.g., a 50% opacity overlay would produce a texture that is half-human and half-animal). In order to generate the human-animal morph stimuli, we therefore used morphological transformation and texture opacity values in Poser to create stepwise morphs from one model to another. These morphs represented the following ratios: 0-animal/100-human, 15/85, 30/70, 50/50, 70/30, 85/15, 100/0. In order to create the human-robot morph stimuli, a slightly different approach was taken. Specifically, we used rendered images of Poser models (i.e., the same human model as before, and *KlanK* from daz3d.com), and entered these into FantaMorph software to create a morph sequence. This change was necessary because we were unable to find a suitable robot morph package for Poser that was compatible with the human figure. The human-robot morph stimuli were generated to represent the same ratios as the human-animal stimuli. All stimuli were cropped and saved as JPEG images at a resolution of 912 × 805 pixels.

A pilot study was conducted to ensure that subjective perceptions of the resulting stimuli were consistent with their objective location on the corresponding continuum. In this pilot study, seven participants rated the 35 stimuli, which were presented in randomized order in a single block, on a 7-point Likert scale ranging from “human-like” to “animal-like” or from “human-like” to “robot-like.” Results indicated that the point of maximal perceptual ambiguity for these stimulus sets was the item within one step of the midpoint (i.e., midpoint plus or minus one step) of each continuum. Response averages indicated that these items were explicitly rated as equally belonging to each of the two categories. Our analyses also revealed that each step in the continuum was perceived as a linear change in the category membership of the model, resulting in overall linear trends for categorical-similarity ratings across each morph continuum. This also ensured that the stimulus at the midpoint of each continuum clearly contained visual-category information from both endpoint categories. None of the participants from this pilot-study were utilized in the subsequent affective-rating task.

The testing apparatus and procedures for the affective-rating task were exactly the same as used in Experiment 1, with the exception that participants provided affective ratings for a total of 35 individual stimuli (five sets of seven morphs) in Experiment 2 compared to 21 stimuli in Experiment 1 (three sets of seven drawings).

### Results

**FIGURE 5:**
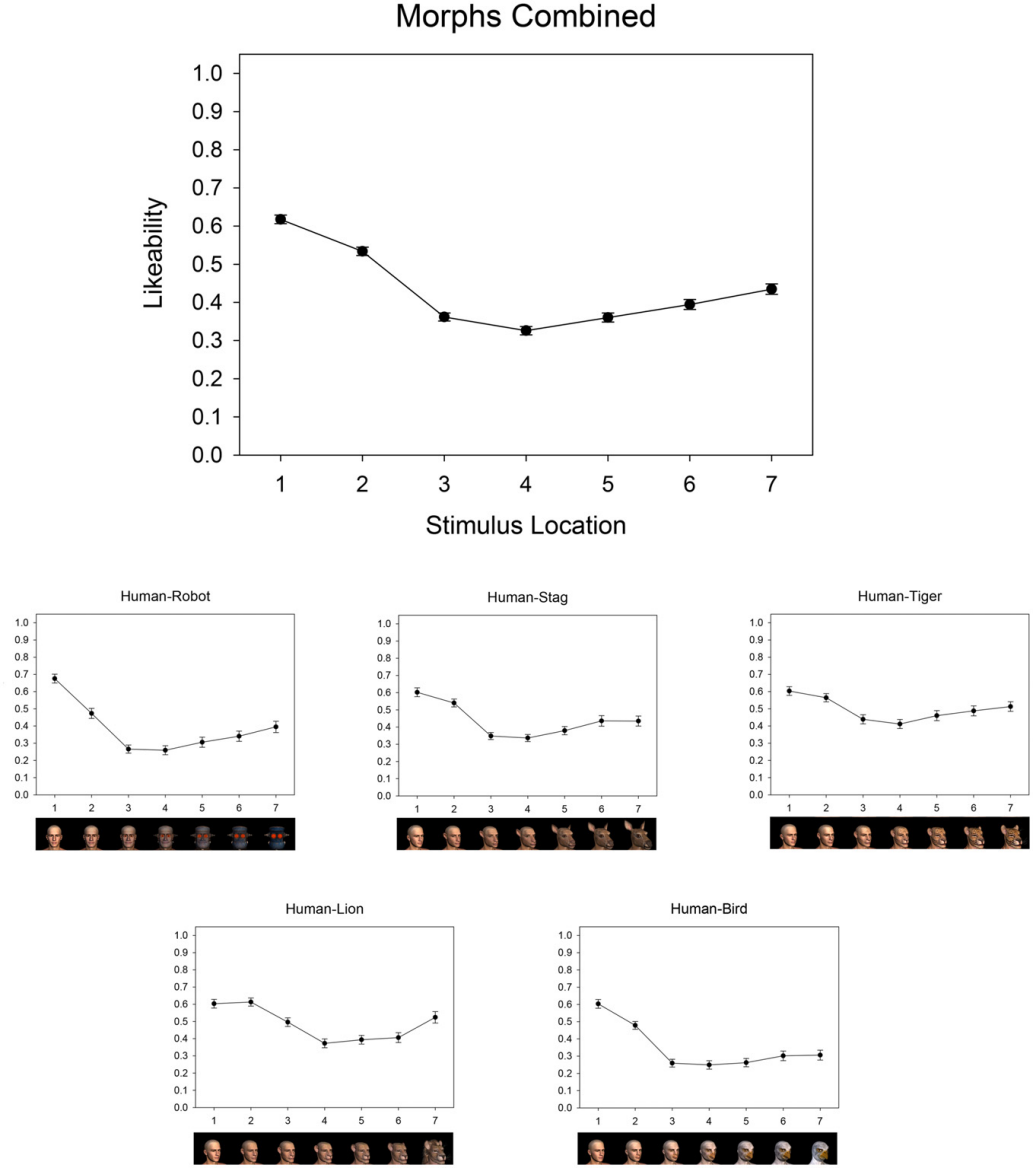
**Affective trends for human-robot and human-animal morphs in Experiment 2**.

Average ratings were calculated for each stimulus as a function of its location along its corresponding continuum. These are plotted separately for each stimulus set in **Figure 5**, along with average ratings calculated across the five stimulus sets.

As a formal test for the existence of an Uncanny Valley function, we again used curve fitting analyses. Raw Akaike values, Akaike Weights, and confidence sets were calculated in order to compare the fit of linear, quadratic, and cubic models.

As indicated by the evidence ratios in **Table 2**, our curve-fit analyses confirmed that non-linear quadratic and cubic models were best fit to the data, whereas linear models fell outside the confidence set. To the extent that such non-linear functions are a defining feature of the Uncanny Valley (Burleigh et al., 2013), this finding is indicative of an Uncanny Valley, not only for the human-robot morph continua, but also for each of the human–animal continua.

**Table 2:** Experiment 2 curve fit analyses.

Set	Model	*Residual sum of squares*	*AICc*	*Δ_i_(AIC)*	*w_i_(AIC)*	*CI*
Human-robot*	Linear^1^	32.69	-1298.74	93.34	0.00	0.09
	Quadratic^2^	27.13	-1386.78	5.29	0.07	–
	Cubic^3^	26.72	-1392.07	0.00	0.93	–
Human-stag*	Linear	23.73	-1453.33	46.15	0.00	0.05
	Quadratic	21.48	-1499.48	0.00	0.47	–
	Cubic	21.38	-1499.74	-0.26	0.53	–
Human-tiger*	Linear	25.78	-1413.40	22.25	0.00	0.03
	Quadratic	24.52	-1435.65	0.00	0.70	–
	Cubic	24.50	-1433.94	1.71	0.30	–
Human-lion*	Linear	26.66	-1452.02	42.92	0.00	0.10
	Quadratic	24.86	-1484.60	10.34	0.01	–
	Cubic	24.25	-1494.94	0.00	0.99	–
Human-bird*	Linear	24.82	-1431.75	67.46	0.00	0.07
	Quadratic	21.58	-1497.26	1.95	0.27	–
	Cubic	21.41	-1499.21	0.00	0.73	–

^1^*K* = 1, ^2^*K* = 2, ^3^*K* = 3, *n = 483.

While the ability to demonstrate an Uncanny Valley for various sets of stimuli is an important manipulation check, our key prediction in Experiment 2 concerned the continuum-location for each stimulus set that received the most negative affective ratings. As shown in **Figure 5**, this valley low-point was located at position 4—the continua midpoint— for each of the human-robot and human-animal stimulus sets (human-robot: *M* = 0.26, SD = 0.22; human-stag: *M* = 0.33, SD = 0.17; human-tiger: *M* = 0.41, SD = 0.22, human-lion: *M* = 0.37, SD = 0.21; human-bird: *M* = 0.25, SD = 0.20). Moreover, paired-samples *t*-tests revealed that average ratings for items near these continua midpoints were indeed significantly lower than those for endpoint items for both the human-robot stimuli (endpoints, *M* = 0.54, SD = 0.17; midpoint, *M* = 0.26, SD = 0.22; *t*(68) = 12.95, *p* < 0.001) and the human–animal stimuli [endpoints, *M* = 0.52, SD = 0.16; midpoint, *M* = 0.34, SD = 0.24; *t*(68) = 9.83, *p* < 0.001].

**FIGURE 6:**
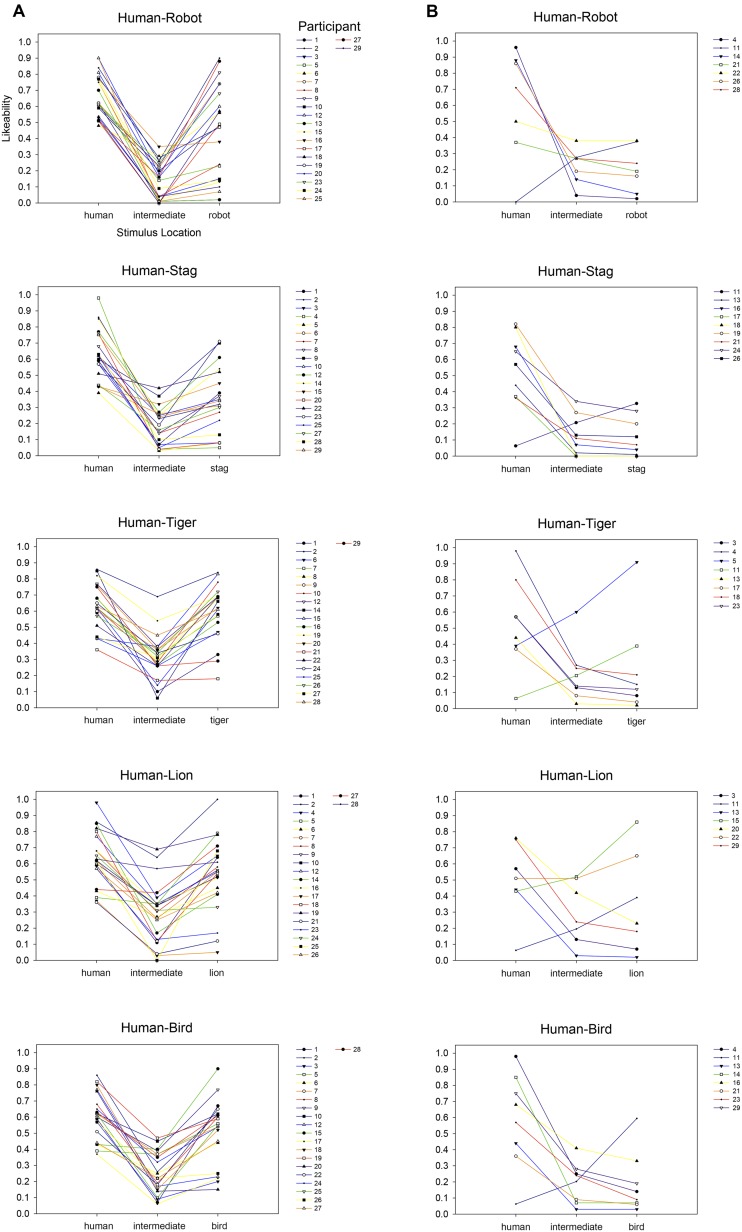
**Individual participants’ affective response to each human-robot and human-animal morph continua in Experiment 2.** Abbreviated rating functions are each comprised of a specific participant’s rating of the stimulus at each endpoint (i.e., positions 1 and 7) along with the lowest rating they provided to an intermediate stimulus (i.e., among positions 2–6). The majority of participants **(A)** provided their lowest affective rating to a stimulus from an intermediate position. The resulting ‘valley’ shape of these individual rating functions is consistent with the Uncanny Valley-type effects identified in the group-average results, but is absent in a minority of participants **(B)** who each provided their lowest rating to at least one of the continua-endpoint items.

As in Experiment 1, we plotted an abbreviated rating function for each participant’s affective response to each perceptual continuum comprised of their rating of each endpoint stimulus along with their lowest rating to an intermediate stimulus. As shown in **Figure 6A**, for each continuum, the majority of participants (79% for human-bird, 76% for human-stag, 76% for human-lion, 72% for human-tiger, and 79% for human-robot) provided their most negative affective rating in response to an intermediate stimulus. The resulting ‘valley’ shape of these individuals’ rating functions is visually evident for each continuum. However, a corresponding minority of participants again provided their lowest rating for an endpoint stimulus, failing to show a valley shape in their individual rating functions (see **Figure 6B**). Indeed, certain participants consistently failed to show Uncanny Valley effects for most (e.g., Participant 4) or all (e.g., Participant 11) morph continua.

Taken together, the results of Experiment 2 replicate previous findings (e.g., Burleigh et al., 2013) and are consistent with the possibility that the affective low-points in Uncanny Valley functions are not determined by the human-ness of the stimuli *per se*, but instead by the amount of conflicting stimulus information during recognition and the need to inhibit competing visual-category information to select one stimulus interpretation over another.

### Assessing Feasibility: Discussion

The data reported above were collected as a first step in experimentally assessing the feasibility of the hypothesis that the Uncanny Valley reflects a form of inhibitory stimulus devaluation. For a perceptual continuum where the endpoints are comprised of two separate categories, this account predicts that maximum negative affect occurs where there is greatest activation of multiple, competing stimulus representations. We conducted two experiments to test the corresponding predictions (1) that the Uncanny Valley also occurs when humans view distinctly non-human stimuli, and (2) that the lowest point of the ‘valley’ does not always occur on the ‘human’ side of the perceptual continuum. In Experiment 1, we used distinctly non-human stimuli: bistable line drawings of animals, a type of stimulus that activates multiple, competing stimulus interpretations. We found that affective ratings of these bistable continua were best fit by non-linear models, which is consistent with an Uncanny Valley interpretation, and that stimuli near the midpoint were rated as least likeable. In Experiment 2, we generated 3D computer-modeled images that comprised human-to-robot and human-to-animal morphed continua. We found that affective ratings of these human-non-human morph continua were best fit by non-linear models, and that stimuli near the midpoint were rated as least likeable. Importantly, we observed that affective minima—the stimulus with the lowest affect rating in each stimulus set—were not always on the ‘human’ side of the human-non-human continua. Taken together, these results are consistent with a general recognition-related account based on the novel hypothesis that the Uncanny Valley is a specific instance of a more general form of stimulus devaluation that occurs when inhibition is triggered to resolve conflict between competing stimulus-related representations.

Mori’s (1970) original hypothesis suggested that near-perfect human-likeness was key to the Uncanny Valley effect, and theoretical accounts such as the pathogen avoidance hypothesis (e.g., MacDorman et al., 2009) were consistent with this premise. These accounts make two basic predictions: the first is that the Uncanny Valley effect should only occur when individuals view stimuli that portray conspecifics (e.g., humans viewing humans), and the second is that the location of the ‘valley’ should be on the ‘human’ side of a ‘human’/‘non-human’ continuum. Our results are consistent with a different interpretation. We found an Uncanny Valley effect with distinctly non-human stimuli. Moreover, when using human-like morph stimuli, the location of the ‘valley’ was not consistently on the ‘human’ side of the continuum. We suggest instead that the Uncanny Valley may reflect the affective consequences of cognitive processes applied to stimuli whose perception strongly activates multiple, competing stimulus representations. This perspective is based on a well-established and growing body of evidence that cognitive inhibition—known for its role in resolving potential interference during visual tasks—has distinctly negative affective consequences for associated stimuli (Fenske and Raymond, 2006; Frischen et al., 2012). Negative affect for stimuli within an Uncanny Valley context may therefore occur to the extent that selecting one stimulus interpretation over the other requires inhibition of visual-category information associated with the non-selected interpretation. The greater the inhibition during identification, the greater the negative affect for the associated stimulus. Importantly, our results here suggest that such stimulus devaluation characteristic of the Uncanny Valley does not depend on the human-ness of the stimuli *per se*.

## Outstanding Issues and Future Directions

Our consideration of prior Uncanny Valley findings and recent advances in our understanding of the affective consequences of cognitive inhibition have led to the hypothesis that affective devaluation by stimulus-related inhibition may underlie the Uncanny Valley effect. The consistency of the results of our preliminary experimental tests of key predictions arising from this new account further establish its feasibility. However, some outstanding issues need to be addressed before the value of inhibitory devaluation can be fully realized as an explanatory construct in Uncanny Valley research.

## Negative Affect from Inhibition or Reduced Fluency?

Yamada et al. (2012, 2013) have suggested that the Uncanny Valley effect may be due to low processing fluency (i.e., the ease with which stimulus-information is processed) for items that are difficult to categorize (e.g., as human or non-human). This account relies on the well-established connection between increases in processing fluency and the experience of positive affect toward associated stimuli (see Reber et al., 2004 for review). There are some reasons, however, to suspect that the distinctly negative responses associated with the Uncanny Valley may be linked to cognitive inhibition rather than fluctuations in fluency, *per se*. First, it is conceivable that a stimulus-processing episode involving inhibition might reduce fluency and the positive affect associated with the item—in which case, processing fluency would be a *proxy* for cognitive inhibition. Second, the affective consequences of fluency are thought to be distinctively positive (Reber et al., 1998; Winkielman and Cacioppo, 2001). Furthermore, experimental conditions that typically favor increased perceptual fluency (i.e., repeated and longer-duration stimulus exposures, and stimulus presentations at central foveal locations associated with high visual acuity) nevertheless lead to distinctly negative affective stimulus ratings whenever successful task performance requires attentional or response-related inhibition (Fenske et al., 2004; Raymond et al., 2005; Frischen et al., 2012). Thus, the available evidence to date points to a clearer link between inhibition and aversive stimulus response than between changes in fluency and stimulus-related negative affect.

### The Challenge of Indirect Measures of Inhibition

We conducted two experiments to assess the feasibility of the hypothesis that the Uncanny Valley reflects the negative affective consequences of cognitive inhibition, yet neither experiment included a measure of inhibition, *per se.* And while the link between inhibition and stimulus devaluation is sufficiently strong that researchers have begun to take the occurrence of increasingly negative subjective stimulus evaluations as a key indicator of the potential involvement of inhibition at key points within a given task (e.g., Kihara et al., 2011), there are certainly many other factors that can lead to an aversive response. Thus, one of the outstanding challenges for further assessing the feasibility of the inhibitory-devaluation hypothesis is to obtain converging evidence that competition between the multiple stimulus-category representations activated by Uncanny-type stimuli is indeed resolved through inhibition of the non-selected representations. Part of the challenge arises from the fact that traditional cognitive-behavioral measures of inhibition (e.g., perceptual response time and accuracy) are also indirect measures—indices of inhibitory aftereffects rather than a metric of inhibition itself. These traditional measures, as well subjective affective ratings, can therefore be influenced by other factors that may systematically accompany inhibition, such as cognitive conflict. Nevertheless, the absence of a direct behavioral measure of inhibition has not precluded the usefulness of using indirect measures in exploring its potential involvement in a wide variety of cognitive faculties (for review, see Bari and Robbins, 2013). Advances in combining cognitive methods with neuroimaging techniques can also provide a converging-methods approach that may be critical for disentangling the specific cognitive and affective sequence of events involved in inhibitory devaluation and the extent to which they contribute to the Uncanny Valley effect. We outline some of these possibilities below.

### Neurocognitive Mechanisms

If the Uncanny Valley effect reflects inhibitory devaluation of stimuli that activate multiple, competing interpretations during recognition, then neuroimaging investigations of the Uncanny Valley should be expected to show critical similarities with neuroimaging investigations of inhibitory devaluation. So far, the only examinations of the neural correlates of inhibitory devaluation include a pair of electrophysiological (event-related potential) studies by Kiss et al. (2007, 2008), and a functional magnetic resonance imaging (fMRI) study by Doallo et al. (2012). Nevertheless, each of these studies have indicated that the magnitude of neural activation associated with resolving potential interference among competing stimulus/motor-response representations are linked to subsequent levels of negative subjective evaluations of the associated stimuli.

Doallo et al. (2012), for example, found that the level of activity in lateral prefrontal cortex (middle frontal gyrus) was greatest during periods requiring response inhibition for successful task performance. The level of activity in this inhibition-related region was linked to the subsequent magnitude of affective devaluation in participants’ subjective ratings of the stimuli. Within the realm of Uncanny Valley effects, Saygin et al. (2012) likewise observed a greater change in activity within a region of middle frontal gyrus (among other lateral areas of the parietal and temporal cortices) when participants repeatedly observed a robot with human features (i.e., a stimulus depicting multiple, competing categories) than when participants repeatedly viewed a robot without human features or a real human (i.e., stimuli that depicted a single object category). It should be noted, however, that Saygin et al. (2012) did not measure affective responses to their stimuli. Nevertheless, some broadly consistent findings across paradigms have also been obtained in fronto-limbic areas thought to be involved in the coding of items’ motivational or emotional significance. Doallo et al. (2012), for example, reported that the level of activity in orbital-frontal cortex, insular cortex, and amygdala during periods of motor-inhibition was linked to the subsequent magnitude of stimulus devaluation. Cheetham et al. (2011) likewise observed relative increases in activity within amygdala and insular cortex when a morph image bearing a greater resemblance to an inanimate human-like avatar was quickly followed by a different image depicting a real human than when followed by another avatar. Their findings support the idea that conditions that evoke multiple, competing stimulus representations can elicit activity in emotion-related areas, even in the absence of motor-related conflict or categorical ambiguity, *per se*. Unfortunately, their passive-viewing approach meant that participants in their study were not asked to provide any explicit perceptual or (even more importantly here) affective judgments about the stimuli they viewed, making it impossible to link the changes in neural activity they observed to subjective perceptual or affective outcomes. And while comparisons are otherwise limited by the many differences in methods and procedures, the consistency in the results of these prior neuroimaging studies of inhibitory devaluation and Uncanny Valley effects certainly does support a call for future studies to directly examine specific issues regarding the extent to which the Uncanny Valley effect reflects inhibitory devaluation of stimuli that activate multiple, competing stimulus representations.

For example, tasks that involve cognitive conflict are thought to rely on the anterior cingulate cortex for conflict detection (Botvinick et al., 2001; Kerns et al., 2004), while the lateral prefrontal regions appear to be recruited during subsequent cognitive control (Carter and van Veen, 2007). This may explain why tasks that evoke cognitive dissonance have been shown to engage the dACC as well as the insula (van Veen et al., 2009) and left dorsolateral PFC (Mengarelli et al., 2013), and why activation in these areas has predicted attitude change in line with cognitive dissonance theory. Indeed, more recent evidence (Izuma et al., 2013) suggests that the same region of the dACC is involved in both cognitive dissonance and conflict. Therefore, if the Uncanny Valley effect can be understood as the affective consequence of inhibition applied to reduce cognitive conflict, then we might expect to see anterior cingulate involvement when participants judge stimuli that present conflicting cues to category membership. This can be assessed by combining Uncanny Valley paradigms such as the paradigm used in our experiments with techniques such as fMRI or EEG/ERP. Electrophysiological markers of conflict detection, such as the N2 event-related potential obtained from frontocentral electrodes (directly above the dACC), for example, may be particularly useful for examining the link between conflict and stimulus devaluation in Uncanny Valley paradigms.

One possibility would involve extending the approach used by Kiss et al. (2008) in their EEG/ERP study of inhibitory devaluation that focused on how changes in the amplitude of the N2 component were linked to the magnitude of stimulus devaluation measured thereafter. Using this approach with Uncanny Valley-type stimulus sets would likewise be expected to reveal the largest N2 component, and the most negative affective response, for those stimuli that most strongly activate multiple, competing stimulus interpretations. Experimental priming manipulations might also be used to vary the extent to which a stimulus from a given perceptual continuum would activate multiple, competing stimulus interpretations. For target images selected from the midpoints of human-robot morph sequences, for example, varying whether a preceding prime image is either a human, robot, or from a completely unrelated (control) category should have an impact on behavioral and neuroimaging measures of cognitive conflict, inhibition, and subjective emotional responses to the target images. The anterior midcingulate cortex (aMCC) has also been linked to cognitive control and negative affect (Shackman et al., 2011). Future fMRI investigations may therefore target the aMCC as another potential link between cognitive conflict, the recruitment of cognitive inhibition and negative affect in situations involving the Uncanny Valley and other conditions known to produce inhibitory devaluation.

### Individual Differences

Another important avenue of research concerns individual differences in inhibitory processes that might explain differing affective responses to ‘uncanny’ stimuli. In our experimental assessment of the feasibility of the inhibitory-devaluation hypothesis (Experiments 1 and 2), we observed that, while the individual affective responses of most participants reflected an Uncanny Valley-like pattern of stimulus evaluation, this was not universal. Individual differences in affective responses to uncanny stimuli could arise either due to varying inhibitory control abilities, or due to variations in the affective sensitivity of an individual to an inhibitory signal. For example, MacDorman and Entezari (2015) recently observed that the eeriness induced by uncanny stimuli was more pronounced among individuals with high trait anxiety. Given evidence that trait-anxious persons exhibit hyper-responsivity in fronto-limbic regions associated with negative affect (Shin and Liberzon, 2009), it is possible that their sensitivity to Uncanny stimuli arises due to heightened affective reactivity to inhibitory signals. Individual differences in the magnitude of other forms of inhibitory-devaluation have also been linked to differences in inhibitory control (e.g., failures to inhibit motor-responses, Ferrey et al., 2012). Exploring the relationship between individual differences in the subjective magnitude of the Uncanny Valley effect and differences in inhibitory control and/or affective sensitivity to inhibitory signals may therefore provide another interesting direction for future research into the specific sequence of cognitive and affective events that the inhibitory-devaluation account of the Uncanny Valley is proposed to comprise.

## Conclusion

The importance of elucidating specific mechanisms underlying the Uncanny Valley effect is underscored by the extent to which interest in this effect has spread from robotics into other areas, such as computer graphics and prosthetics (Seyama and Nagayama, 2007; MacDorman et al., 2009; Mitchell et al., 2011; Tinwell et al., 2011; Poliakoff et al., 2013). Whereas many accounts of this effect have focused on the potential relationship between the subjective human-likeness of a stimulus and an observer’s emotional response to it (e.g., MacDorman and Ishiguro, 2006; Seyama and Nagayama, 2007; MacDorman et al., 2009), we propose an alternate account based on the novel hypothesis that the Uncanny Valley is not directly related to ‘human-likeness’ *per se,* but instead reflects a more general form of stimulus devaluation that occurs when inhibition is triggered to resolve conflict between competing stimulus-related representations. Our preliminary assessment, both in terms of prior findings as well as through our experimental tests of two specific predictions arising from this new account of the Uncanny Valley, establish its feasibility and make clear the need for additional research into the possibility that the strong dislike of Uncanny-type stimuli reflects the negative affective consequences of conflict-resolving inhibition.

## Conflict of Interest Statement

The authors declare that the research was conducted in the absence of any commercial or financial relationships that could be construed as a potential conflict of interest.
